# Morocco's Healthcare System: Achievements, Challenges, and Perspectives

**DOI:** 10.7759/cureus.41143

**Published:** 2023-06-29

**Authors:** Mohamed Mahdaoui, Najib Kissani

**Affiliations:** 1 Neurology Department, Mohammed VI University Hospital Center of Marrakech, Marrakech, MAR

**Keywords:** social coverage, non-communicable diseases epidemiology public health community-based study, global healthcare systems, public health care, morocco

## Abstract

Despite progress in recent years, access to quality medical care remains a significant issue, particularly in rural areas. The unequal distribution of resources, inadequate funding, healthcare worker shortages, and the rise of non-communicable diseases pose substantial challenges. However, implementing universal health coverage and improving key health indicators demonstrate notable achievements. To further enhance the healthcare system, perspectives such as addressing resource disparities, increasing funding and the healthcare workforce, managing non-communicable diseases, embracing digital technologies, strengthening public healthcare, and focusing on prevention and health education are proposed. These perspectives offer a global vision for improving Morocco's healthcare system's efficiency, inclusiveness, and quality, ultimately ensuring equitable access to healthcare services for all citizens.

## Editorial

Introduction

The healthcare system is an essential component of any society, as it plays a crucial role in maintaining the health and well-being of its citizens. Like many other countries, the healthcare system faces various challenges in Morocco. Despite efforts to improve healthcare, access to quality medical care remains a significant issue, particularly in rural areas. This article explores the challenges facing the Moroccan healthcare system, such as funding and resource allocation, and the achievements made in recent years, such as the implementation of universal health coverage. Through an analysis of these challenges and triumphs, we can gain a better understanding of the strengths and weaknesses of the Moroccan healthcare system and identify areas for improvement.

However, recent achievements in the Moroccan healthcare system offer hope for a brighter future, and several perspectives can guide future efforts to incorporate perspectives from global healthcare systems, Morocco can further enhance its healthcare system and strive towards international standards and best practices.

Achievements in the Moroccan healthcare system

Despite the challenges facing the Moroccan healthcare system, there have been several notable achievements in recent years that offer hope for improvement. One significant achievement is the implementation of universal health coverage through the Ramed program in 2011. This program aims to provide health coverage to vulnerable populations, including those who are poor or have chronic diseases.

The Ramed was replaced in 2023 by the generalization of social coverage for the benefit of all Moroccans, which was launched on July 29, 2020, by King Mohammed VI. This project requires a rigorous reform of social systems and programs following the crisis linked to the COVID pandemic, which has highlighted several shortcomings, particularly the weakness of social protection networks [1].

Over the years, there have been significant improvements in key health indicators. Life expectancy at birth has seen a remarkable rise, increasing from 47 years in 1967 to 74.8 years in 2013. Infant mortality rates have seen a substantial decline, with numbers falling from 113.6 per thousand live births in 1967 to 28.8 in 2013. Similarly, maternal mortality has witnessed a significant decrease, dropping from 359 per hundred thousand live births in 1981 to 112 in 2013 [2].

The healthcare system has also made great strides in vaccination coverage, with a commendable 94.5% coverage rate and equitable distribution across different regions [2]. This has resulted in the elimination of targeted diseases like poliomyelitis and diphtheria, as well as a notable decrease in the incidence of diseases such as measles [3].

The expansion of health coverage has been a priority, evident through a substantial increase in the number of basic healthcare establishments and hospitals throughout the country. Moreover, there has been a notable rise in the number of doctors, with a ratio of 1 doctor per 1775 inhabitants in 2009, which improved to 1.5 doctors per 1000 inhabitants in 2014 [2].

Another significant achievement is the development of the pharmaceutical industry in Morocco. The industry has been successful in meeting 70% of the national demand for drugs, ensuring a more self-sufficient and accessible healthcare system for the population.

These accomplishments in Moroccan healthcare reflect the country's commitment to improving the well-being of its citizens and provide a solid foundation for the continued growth and advancement of the healthcare sector.

The Moroccan government has also invested in expanding healthcare infrastructure, including building new hospitals and clinics and upgrading existing facilities. Additionally, several e-health services, such as telemedicine and electronic health records, have been developed to improve healthcare access and efficiency [2].

Challenges facing the Moroccan healthcare system

Despite significant progress in recent years, the Moroccan healthcare system faces several challenges that limit its ability to deliver high-quality healthcare to all citizens. One significant challenge is the unequal distribution of healthcare resources, with urban areas having better healthcare access and resources than rural areas. This disparity is particularly pronounced in the areas of healthcare infrastructure, medical equipment, and specialist care [1].

Another significant challenge is inadequate funding for healthcare, which limits the ability to provide essential medicines and technologies, improve healthcare infrastructure, and increase healthcare access. Additionally, there is a shortage of healthcare workers, particularly doctors and nurses, especially in rural areas. This shortage results in longer wait times for patients and limited access to specialist care [1].

Furthermore, non-communicable diseases (NCDs) are increasingly prevalent in Morocco, posing a significant health burden [4]. NCDs account for over 80% of deaths in Morocco, and the healthcare system is not adequately equipped to manage these diseases. The prevalence of risk factors for NCDs, such as tobacco use, unhealthy diets, and physical inactivity, is also high in Morocco [4].

Moreover, insufficient health insurance coverage: 38% of the population does not have medical coverage, and those who do have coverage may still face high out-of-pocket expenses for healthcare services [1].

The Moroccan healthcare system struggles with the management of health data and information, which limits its ability to make informed decisions, monitor healthcare quality, and plan healthcare strategies [1].

Finally, we can see that the main source of these difficulties is generally the lack of optimal, rational, and effective governance; good governance has become an essential factor in efforts to strengthen health systems and its impact on health is now more decisive. The overall governance of the system does not empower the actors and does not encourage quality [1,5].

Perspectives for the future of the Moroccan healthcare system

While the recent achievements in the Moroccan healthcare system are promising, several perspectives can guide future efforts to improve the country's healthcare system.

The health map project which is currently being carried out aims to organize the care pathway territorially, to lighten the load on health structures, reduce waiting times and properly distribute care between regions [1].

In this scheme, the university hospital center plays an essential role as a leading specialty, research, and training institution and also as a structure to which the other hospitals in the region are linked within the framework of the Regional Health Groups.

The Public-Private Partnership is another means that aims to improve health services for the population, which will also make it possible to pool the resources of the public and private sectors and allow, for example, the health professionals of the latter to offer health care at the level of public health structures by taking advantage of their technology platform.

Addressing the unequal distribution of healthcare resources between urban and rural areas is essential. To achieve this, there must be a concerted effort to invest in rural healthcare infrastructure, medical equipment, and specialist care, as well as increase the number of healthcare workers in these areas [1].

Adequate funding for healthcare is necessary to provide essential medicines, medical equipment, and technologies, and improve healthcare infrastructure. Increased funding will also facilitate the recruitment and retention of healthcare workers, particularly in rural areas.

Managing and preventing NCDs should be a priority. This requires a comprehensive approach that includes promoting healthy behaviors, improving healthcare access and quality, and addressing social determinants of health that contribute to the development of NCDs [2].

There are several propositions to enhance the Moroccan health system and pave the way for further improvements. First, the digitization of the health system can bring significant benefits. By embracing digital technologies, such as electronic health records and telemedicine, healthcare delivery can be streamlined, ensuring efficient management of patient data and enabling remote consultations. Additionally, reorganizing the care pathway from the community level to the regional level can improve access and coordination of healthcare services, allowing for better integration and collaboration among healthcare providers.

To strengthen the public healthcare sector, efforts should be made to make public hospitals more attractive. This can be achieved by empowering them to generate their resources, promoting financial sustainability, and implementing measures that enhance the quality and efficiency of services. Encouraging public-private cooperation can also play a vital role in leveraging the strengths of both sectors, fostering innovation, and expanding access to healthcare for all.

Another important perspective is the implementation of an integrated and intersectoral policy focused on prevention and health education. By addressing health promotion and prevention strategies, such as disease prevention campaigns and education programs, the burden of illness can be reduced, and individuals can be empowered to make informed choices about their health. This requires collaboration across different sectors, including education, public health, and community organizations, to create a comprehensive and effective approach to improving population health.

By embracing these propositions and perspectives, Morocco can lay the foundation for a more efficient, inclusive, and patient-centered health system that prioritizes prevention, embraces innovation, and ensures equitable access to quality healthcare services for all its citizens.

Conclusion

The Moroccan healthcare system faces challenges such as resource disparities, inadequate funding, and non-communicable diseases. However, achievements like universal health coverage and improvements in health indicators show progress. To enhance the system, addressing resource disparities, increasing funding and workforce, managing non-communicable diseases, embracing digital technologies, and focusing on prevention are essential. These steps can pave the way for an efficient, inclusive, and patient-centered healthcare system in Morocco.
